# Improved safety standards are needed to better protect younger children at playgrounds

**DOI:** 10.1038/s41598-018-33393-z

**Published:** 2018-10-10

**Authors:** Xiaogai Li, Svein Kleiven

**Affiliations:** Division of Neuronic Engineering, Department of Biomedical Engineering and Health Systems, KTH Royal Institute of Technology, Huddinge, 141 52 Sweden

## Abstract

Playground-related traumatic brain injuries (TBIs) in children remain a considerable problem world-wide and current safety standards are being questioned due to historical reasons where the injury thresholds had been perpetuated from automobile industry. Here we investigated head injury mechanisms due to falls on playgrounds using a previously developed and validated age-scalable and positionable whole body child model impacted at front, back and side of the head simulating head-first falls from 1.59 meters (m). The results show that a playground material passing the current testing standards (HIC < 1000 and resultant linear acceleration <200 g) resulted in maximum strain in the brain higher than known injury thresholds, thus not offering sufficient protection especially for younger children. The analysis highlights the age dependence of head injuries in children due to playground falls and the youngest have a higher risk of brain injury and skull fracture. Further, the results provide the first biomechanical evidence guiding age-dependent injury thresholds for playground testing standards. The results also have direct implications for novel designs of playground materials for a better protection of children from TBIs. Only making the playground material thicker and more compliant is not sufficient. This study represents the first initiative of using full body human body models of children as a new tool to improve playground testing standards and to better protect the children at playgrounds.

## Introduction

Playgrounds have social and physical benefits for children, but these settings also pose a threat of injuries, especially traumatic brain injuries (TBIs) not only lead to substantial financial burden but also long lasting consequences for the victims. Children being injured at playgrounds are a common and global health problem and fall to the playground surface is reported to be a major cause^1–4^. A recent study shows that playground-related TBIs have risen significantly over the past decade from 18,629 in 2001 to 29,514 in 2013 in the U.S., where most of the children are between 5–9 years old^1^. While in Sweden, an estimated 16,000 children every year receive an emergency reception for playground-related injury, most injured age group is between 4–6 years old and TBIs accounted for almost 20%^2^. These numbers suggest continuous effort and strategies are needed to reduce playground injuries.

To reduce the risks of serious and fatal injuries from falls, a number of playground safety standards have been established. ASTM F1292^5^ is the impact attenuation standard established in 2004 being used in the U.S. and EN 1177^6^ is the equivalent impact attenuation standard used in a number of European countries starting from 1998. The above mentioned safety standards are intended to reduce the injury risks for the head and the protection is evaluated by measuring the accelerations of a hemispherical metal missile (e.g. ASTM F1292 uses a metal hemispherical mass of 4.6 kg simulating the head of a child). Current standard requires a Head Injury Criteria (HIC) score lower than 1000 and a peak resultant linear acceleration not exceeding 200 g (gravities) when the missile is dropped from a critical height. Worth noting that these tolerance values had been perpetuated from research results performed in the field of automobile industry^7^. The question is: Does the same global head injury threshold obtained in automobile industry apply to children injured at playground due to falls when transferring to brain tissue injury?

Complicating the matter further is the advances in the understanding of injuries in automobile industry during the 1990s have led to updated HIC thresholds (700 for 6 years old (YO) and above, 570 for 3YO and 390 for 1YO)^8^ which have become active in the automobile industrial standard. Due to historical reasons being playground thresholds adopted from the automobile industry, the update in automobile industry brings a question: Should playground testing standards be updated as well, and should the same age-dependent HIC apply? Currently, there is very limited evidence for advocating such changes except epidemiological studies, which are however complicated by many factors and makes the evidence unconvincing. Besides the knowledge gaps by adopting injury thresholds directly from automobile industry, playground surface testing procedures are much more simplified by dropping a hemispherical metal missile^5^, far less developed than in automobile industry or helmet industry where dummy heads with improved biofedelity are being used.

Comparing with the numerous experimental and computational studies on understanding TBIs in automobile industry and sport^9–17^, efforts on playground-related TBIs in children are far lagging behind. The efforts both research-wise and economically on automobile safety indeed has led to a significant decrease of TBIs in traffic accidents during the last decades. On the contrary, playground-related TBIs remain at a constant level^2^ or are even increasing^1^. The effectiveness of playground safety standards is being questioned and research efforts are urged to be brought into action^4,7,18–20^.

There is a lack of studies on TBIs in children falling to playgrounds, despite relevant studies exist which either focus only on playground materials^21^ or limited to household falls using anthropomorphic test devices (ATDs)^22–26^. ATDs indeed provide valuable insights into the global impact kinematics of the head, but doesn’t allow for predicting tissue level injury. Finite element (FE) human body models (HBMs), have the potential to provide significant insights into the response to impact. Compared with ATDs, HBMs can accurately represent the complex anatomy of the human body and the growth with age, thus allow assessment of local mechanical behaviors and estimation of human tolerances to external forces. Validated models are playing an increasing role in evaluating safety designs.

The Position and Personalize Advanced Human Body Models for Injury Prediction (PIPER) child model together with a PIPER tool developed in an European project enables a continuously scalable approach to perform computer simulations with HBMs of different ages, different sizes for a given age, or with HBMs in different positions, starting from a baseline model^27^. The model responses have been compared to experimental studies for all body regions, showing a good performance including: drop and compression tests for the head; bending and tensile tests for the cervical spine; pendulum and belt interaction tests for the trunk; bending tests for the lower extremities and full body sled tests for the mobility of the spine; side impacts for shoulder and pelvis^27^. The model has been used in a variety of applications such as vehicle accident reconstructions^15^ and studying cervical spine injuries^28^, showing promising results.

The objective of this study is to use the PIPER model to evaluate whether or not current playground testing standards provide sufficient protection for children’s brain. Further we hypothesized that an age-dependent risk of head injuries exists from playground falls. The PIPER scalable HBM in combination with the PIPER tool are used to scale the baseline PIPER model to different ages from 1.5 to 18 years old. A series of fall simulations impacted at a baselinie playground material at three different locations of the head (front, back and side) are investigated. HIC values, linear and rotational global kinematics at the centre of gravity (C.G.) of the head are extracted to evaluate head injury risks according to the current testing standards. Further, tissue injury parameters in terms of skull stress and brain strain are analysed.

## Results

### Impact kinematics during falls

**Figure 1:**
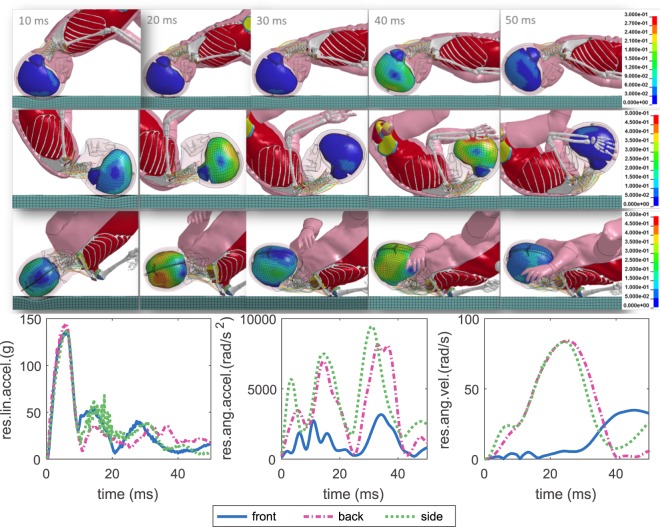
Simulated full body impact kinematics with 1^st^ principal G-L brain strain visualized for front (row 1), side (row 2) and back impacts (row 3), illustrated with the 3YO impacted at a baseline playground material. Time-history curves of res.lin.accel (left), res.ang.accel. (middle) and res.ang.vel. (right) are extracted at the C.G of the head for the three impact directions (lower row).

The full body kinematics at front, back and side directions when impacted to a baseline playground material are illustrated with the 3YO model (Fig. 1). Additional animations showing the entire dynamic impact response for each impact are provided as Supplementary Videos. The time-history curves of resultant linear accelerations (res.lin.accel.) at the C.G. of the head are similar between different impact directions and the peak values are comparable despite slightly higher for a back impact. A much larger difference is seen in the resultant angular acceleration (res.ang.accel.), front being much lower than side and back. Similarly, the resultant angular velocity (res.ang.vel.) of frontal impact is much lower than the other two directions (Fig. 1, lower row). Time-history curves of other ages show similar characteristics (see Supplementary Results).

### Age-dependent head injury kinematics

**Figure 2:**
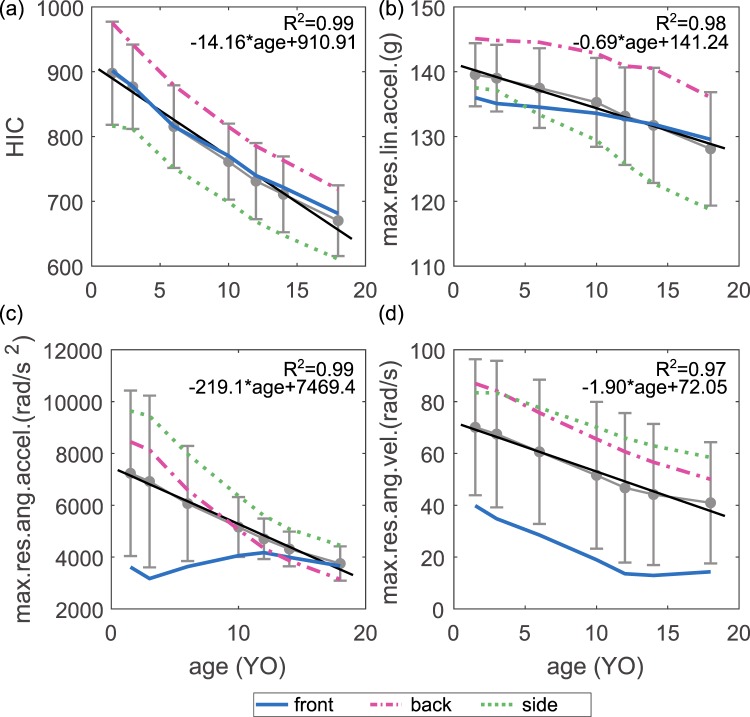
Linear regression of the average of the three impact directions showing age-dependence of (**a**) HIC, (**b**) maximum res.lin.accel., (**c**) maximum res.ang.accel., and (**d**) maximum res.ang.vel. Error bars are plotted together with the average values between front, back and side impact.

Time-history curves of res.lin.accel., res.ang.accel. and res.ang.vel. for all three impact directions are extracted for all ages (1.5, 3, 6, 10, 12, 14, 18YO) and only the peak values are presented, as well as HIC as a function of age (Fig. 2). The average of all the evaluated parameters for the three impact all shows a linear decrease with age (R^2^ all above 0.97) (Fig. 2), indicating a younger child has a higher risk of head injury if we assume the same kinematic injury threshold for all ages.

The youngest age (1.5 YO) back impact results a largest HIC of 975.3 among all ages and all impact directions, corresponding to a max.res.lin.accel of 145.1 g (Fig. 2). Of interest note this result is consistent with previous tests of playground materials using hemispherical missile. HIC is more conservative than max.res.lin.accel, and at HIC 1000, max.res.lin.accel is far from reaching the threshold of 200 g. Further, the side impact of an 18YO has a lowest HIC of 610.9 with a lowest acceleration of 118.7 g.

**Figure 3:**
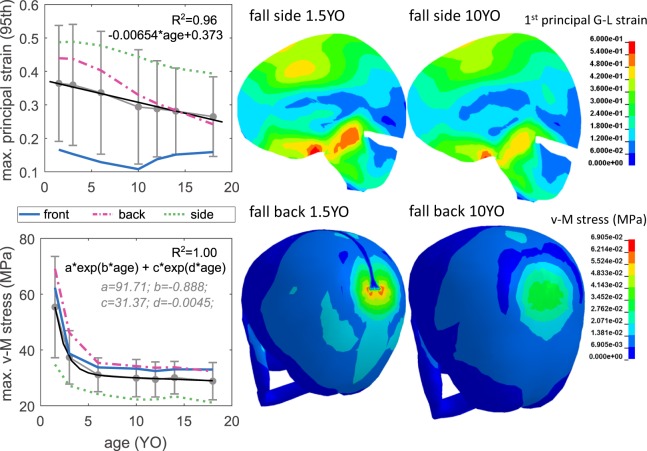
Age-dependence of MPS in the brain and maximum v-M stress in the skull (left). A sagittal plane of brain strain (1.5 and 10YO fall side) and skull stress (1.5 and 10YO fall back) captured when peak value occurs (right).

The impact direction has a smaller influence on the linear-based kinematics (HIC and linear acceleration) compared with rotational acceleration and velocity. For example, HIC and max.res.lin.accel for a 3YO front, back and side impact are 877.3, 941.7, 811.5 and 135.1 g, 144.8 g, 137.1 g, resulting a maximum difference of 13.8% and 6.7% between different directions respectively. While a much larger influence is observed for rotational acceleration and velocity, being 66.5% and 58.3% respectively between all impact directions for a 3YO. The influence of impact direction has a similar trend for other ages. The large influence of the impact direction on the rotational kinematics transfers to tissue level as brain strain since the brain is more sensitive to rotational motion (Fig. 3).

### Age dependent brain strains and skull stresses

The 95^th^ percentile maximum 1^st^ principal Green-Lagrangian (G-L) strain (referred to as MPS) in the brain is much lower in a front impact compared with the other two directions (Fig. 3, upper row). The youngest age 1.5YO has the largest MPS of 0.17, 0.44, 0.48 for front, back and side impact respectively. A linear regression of the average of all impact directions (*R*^2^ = 0.96) shows a decrease of MPS in the older children. The average MPS for all impact directions for a 1.5YO is 0.36 compared within the brain for the 10YO of 0.29, showing a decrease of 19.3% in MPS. The distribution of 1^st^ principal G-L strain is illustrated in a sagittal plane for the 1.5YO and 10YO, showing a similar pattern but a larger magnitude in the younger. Moreover, side impacts have the largest MPS in all three directions among all ages.

Side impacts to the head causes smallest peak skull stress while a back impact has the largest for all ages (Fig. 3, lower row). An average of all three impact directions shows a decrease with age following an exponential trend (*R*^2^ = 1.00). The maximum skull stress in the younger ages of 1.5 and 3YO is attributed to the skull anatomy changes with sutures and grows to bones in older children. The largest stress in skulls with sutures occurs at the interface between the suture and the bone (Fig. 3 right).

### Correlation between HIC and other injury predictors

**Figure 4:**
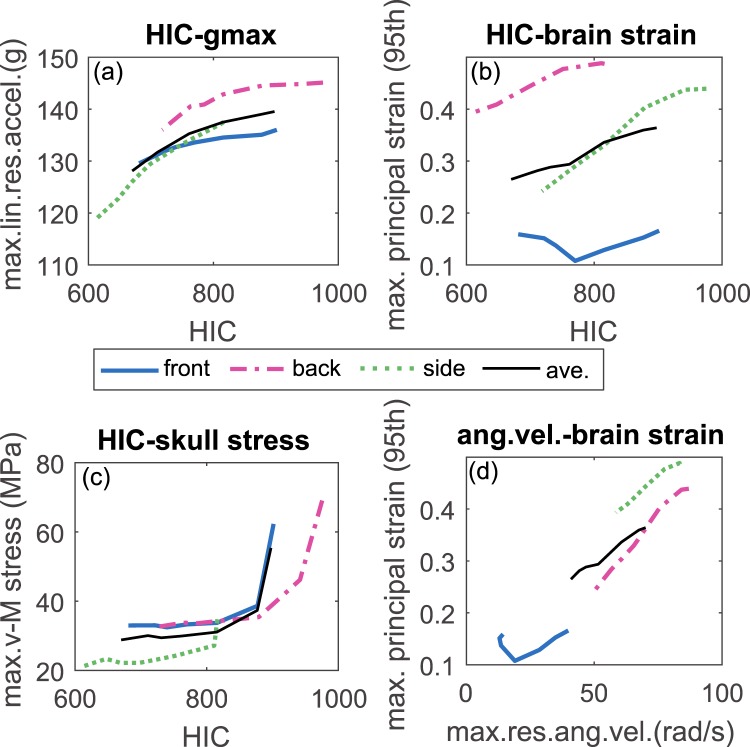
Correlation between HIC and maximum resultant acceleration (**a**), HIC and MPS and the average between all three impact directions shows a linear correlation with *R*^2^ = 0.98 (**b**), HIC and maximum skull stress (**c**), res.ang.vel. and MPS (**d**). The curves for each impact direction as well as the average of the three impact directions are presented.

A larger HIC in general corresponds to a larger value of the evaluated injury predictors (Fig. 4). The correlation between HIC and brain strain when fitted using a linear relation the average between all impact directions leads to HIC being 753.5 for 0.3 in brain strain (Fig. 4b) (corresponds to 50 percent risk of mild TBI^9^. In particular, peak skull stress is nonlinearly increasing with HIC (Fig. 4c) due to the special skull anatomy of the young ages with sutures causing a larger skull stress nearby (Fig. 3). The res.ang.accel., especially the average of the three impact direction increases linearly with MPS (Fig. 4d). The data suggests HIC seems a good indicator for risk of brain injury, but not for skull stress especially for the youngest age.

### Influence of playground stiffness

**Figure 5:**
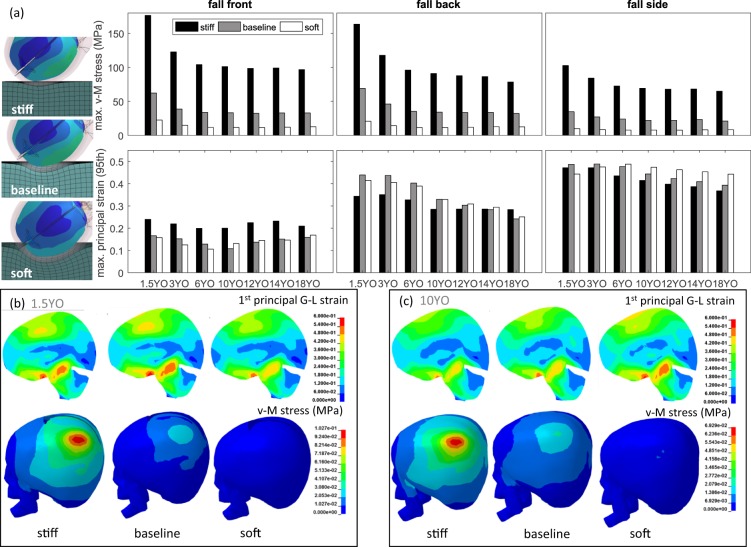
(**a**) Bar plots show the influence of impact direction and playground stiffness on MPS in the brain (row 1) and von-Mises skull stress (row 2). Image on the left captured at maximum indentation depth of the playground to visually illustrate the softness of a baseline the tested stiff and soft playground material, a 1.5YO side impact is shown in the illustration. Sagittal plane shows the distribution of 1^st^ principal G-L strain when maximum value occurs; skull stress distribution captured when maximum value occurs for the 1.5YO side impact (**b**) and the 10YO side impact (**c**).

The results presented above suggest the current standard playground material does not offer sufficient protection to the brain especially for side and back, though front has MPS all lower than 0.2 (Fig. 3). A question is: Can a softer playground protect the brain? Therefore a softer playground material is tested, and for comparison a stiff material is also tested. With the same impact condition, a softer playground material significantly reduces linear acceleration and consequently HIC (see Supplementary Results), which in turn largely reduces skull bone stress, but the MPS in the brain are still above 0.3 especially for a side impact (Fig. 5). For a front impact, a soft material leads to a slight decrease in MPS in smaller ages (1.5YO, 3YO and 6YO), but the older ages do not benefit from a soft material, on the contrary, the MPS is slightly increased unexpectedly. For back and side impact, a soft material in general also leads to unexpected larger MPS than the baseline (Fig. 5a). The 1^st^ principal G-L strain distribution is captured when maximum value occurs, illustrated with a 1.5YO (Fig. 5b) and a 10YO (Fig. 5c) side impact which has largest value of the three directions. The larger MPS caused by a softer material seems to be caused by a rotation allowed during the impact compared with the baseline. The results also highlight that more compliant playgrounds would reduce the risk of skull fractures but not brain injuries.

An analysis of playground stiffness on global parameters including HIC, res.lin.accel, res.ang.accel, and res.ang.vel. for all impacts are found in the supplementary document Supplementary Results. Further, the time interval used for HIC calculation HIC is around 3 ms for a stiff material, and baseline of 6.3 ms compared to a softer material of 14 ms (Supplementary Results). Thus for hard and baseline playground material, HIC15, HIC36, and HIC unlimited are equivalent. While for soft material, the HIC unlimited are only marginally different than HIC15 and HIC36.

## Discussion

The analysis shows a playground material passing the current playground safety standard with a HIC of 1000 and resultant linear acceleration <200 g doesn’t offer sufficient protection to children’s brain. Further, an age-dependent head injury risk is observed, a young child has a higher risk of head injury, both skull fracture and brain injury, supporting an age-dependent injury threshold in playground testing standard. Importantly, the results provide the 1^st^ biomechanical evidence for age-dependent HIC threshold particularly for playground surfacing safety standard. In addition, the results show impact direction has a smaller influence on linear global kinematics (HIC and linear acceleration) but larger influence on the rotational kinematics (rotational acceleration and velocity), and consequently a larger influence on brain injury risks. Lastly, the results show that more compliant playgrounds would reduce the risk of skull fractures but not TBI, which highlight that a more protective playground cannot simply be made to be softer. Instead other innovative designs are to be resorted. The analysis clarifies a few knowledge gaps in the current playground testing standards.

The PIPER child model is shown to be promising for evaluating playground-related head injuries. Indeed the simulated results are in general consistent with the current testing standard. For instance, a playground material with HIC of 1000 leads to a HIC of 975.3 (for the 1.5YO back impact) in the head of the PIPER model. Further, HIC is found to be more conservative than peak linear acceleration (e.g. HIC of 975.3 corresponds to peak g of 145.1 g), same trend as reported when evaluated according to current testing standard using a rigid hemisphere. Obviously compared with the simplified hemisphere, the PIPER model allows more accurate evaluation of injuries due to its higher biofidelity also inclusion of the body better represent real-world falls. Further, the PIPER model allows accounting for anthropometric dimensions of different ages, as well as age dependent material properties.

The evaluated parameters including both global kinematics (Fig. 2) and local tissue response (Fig. 3) indicate a higher risk of injuries in younger child. Thus to protect the younger to the same level as the older ones, age-dependent injury threshold of HIC is suggested for playground safety standards. Based on the correlation between HIC and tissue level injury predictors (Fig. 4), it is possible to propose age-dependent HIC thresholds by using brain strain or skull stress as a scaling factor. For instance, if use the peak skull stress for scaling, the corresponding values are 366, 543, 654 for 1YO, 3YO and 6YO respectively. These values are indeed comparable to the age-dependent values used in automobile industry, being HIC of 390, 570 and 700, which were developed via a combination of FE analysis and scaling techniques^8^. Note above is a simplified calculation based on the average response of falls at three different impact directions, also from a critical height corresponding to a certain type of playground. Falls to the background could be quite complicated in terms of impact direction, impact height and different types of playground. Thus more systematic investigations are needed to develop age-dependent injury threshold of HIC targeting at playground standard. Nevertheless the PIPER model is shown to be promising for developing age-dependent injury threshold for playground falls, which could be different than the one developed in automobile industry.

Conventionally, head impact kinematics are usually investigated with an isolated head or combined with a neck both in numerical models to save computational time, the same holds true in physical test to reduce the complexity. In EuroNCAP rating^29^ for example, linear impacts of isolated dummy head form is used to evaluate the protection to the head. Recent integrated collaboration efforts have resulted into detailed full body models including adult models^16,30,31^ and child models. Despite the use of the full body model allows representing more realistic boundary conditions during impact allows more reliable predictions than an isolated head model, its applicability to be introduced as a tool evaluating playground performance is challenged due to computational cost. Previous studies have shown the body has a prominent effect on impact kinematics and consequently brain injury predictions in windscreen impact for pedestrians^32^ despite a smaller difference in cyclist among adults^33^, and it would be interesting to investigate whether a head only model could be used as a simplification and how much differences in the response compared with inclusion of the whole body under playground falls.

The PIPER model used in this study represents a state-of-art model with major components validated against experimental data^27^ and has been shown promising in studying head and neck injuries during automobile crashes^15,28^. Indeed loadings to the head could be quite different or similar between automobile crashes and playground falls attributing to complicated impact scenarios in both situations. Due to paucity of experimental data, different components of HMBs are usually validated under certain loading condition in terms of loading rate and magnitude. In fact, the global responses in terms of linear acceleration of the PIPER head model have been compared against fall experiments, and brain motion are compared to experimental data under impact loading. Thus, the use of PIEER model for studying playground-related head injuries is thought to be justified.

A free fall from critical height of 1.59 m is simulated in this study to investigate age-dependent head injury risks. As the baseline material represents a playground in common use, simulating a fall from critical height is considered to be a representative loading under which the playground could offer a protection according to the current testing standard (i.e. HIC < 1000). The age-dependent risks of head injuries under different heights, also how playground protects other parts of the body could be investigated in future work. Nevertheless the current results uncover the age-dependent head injury risks to the brain and skull, highlighting the need to protect the most vulnerable and active groups at playgrounds.

Although the PIPER child model has shown promising response compared with experiments in a variety of impact situations, more advanced modelling strategies such as active muscles^16,30^ could be included in the future. Further, injuries in children due to household falls causing a large suffering are also challenging forensic investigators. The promising results from this study suggest a potential of using the PIPER child model for studying injuries occurring in household falls due to its uniqueness for accounting different ages and positions. This allows case-specific investigations by reconstructing falls and assessment of injuries in major organs not limited to the head, thus have a potential to aid forensic diagnosis by providing biomechanical evidence.

## Methods

### PIPER scalable HBM

**Figure 6:**
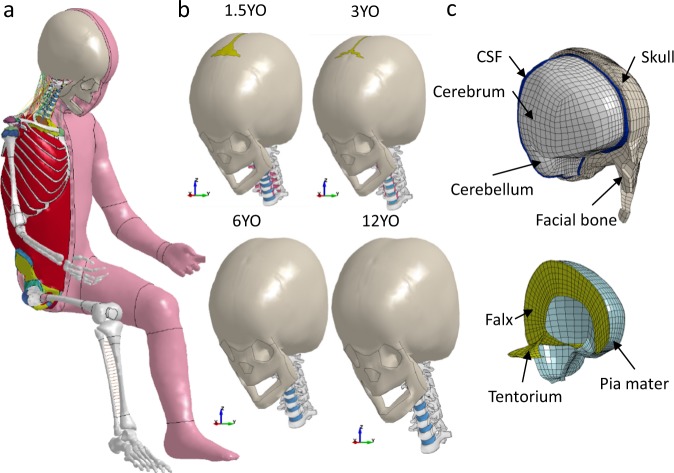
(**a**) The PIPER model is positioned prior to fall simulations, illustrated with a baseline of a 6YO. The same positioning angles are applied to all ages. (**b**) The head and neck models of the 1.5YO, 3YO, 6YO and 12YO are isolated to illustrate continuous growth model accounting the sutures in the head and cartilage in the neck bone. (**c**) The isometric view of the head model showing the skull, brain and facial bone (upper) and the brain is exposed to show the inner membranes of falx, tentorium and the pia mater (lower), illustrated with the baseline head model of a 6YO.

The PIPER scalable HBM^27^ is a detailed child full body model able to describe the growth process and the variation in relevant anatomical regions for children (Fig. 6). The baseline model describes the anatomy of an average 6YO child and has a total mass of 23 Kg. The anthropometric dimensions were normalized by nonlinear scaling using GEBOD^34^ regressions as reference. Overall, the model is composed of approximately 531,000 elements (including about 52,000 rigid elements) distributed into 353 parts describing the main anatomical structures. The model was developed in the LS-Dyna explicit FE code and has a time step of 0.32 µs obtained with marginal mass scaling (15 grams added).

### Age scaling and positioning

The baseline PIPER child model of a 6YO is scaled to 1.5, 3, 6, 10, 12, 14, 18YO using the PIPER tool (v1.0.1), following the same approach as detailed previously^15^. Similarly, all models were positioned using the pre-positioning module of the PIPER tool to position the body parts either by relative frame with respect to the global frame or using model joints. Table 1 shows the angles used to position the child model prior to simulation to mimic a “startled posture” during fall. Unlike previous study^15^ the same material properties were used for different ages, this study incorporates age-dependent material properties for the head and neck to study age-dependent head injury mechanisms. The neck material properties of different ages are according to the data presented earlier^28^, while material properties of different components in the head are presented in the following section. Age-dependent material properties of the remaining parts of the body were generated using the Material Scaling module^35^ implemented in the PIPER tool.

**Table 1:** Angle values used for positioning prior to fall simulations for all ages.

Frame Name (relative to world frame)	Angle (ry)
Atlas	12°
Axis	12°
Third cervical vertebrae	12°
Four cervical vertebrae	12°
Skull	17°
**Joint name**	
Knee	−30°
Hip	−20°
Ankle	15°
Glenohumeral	−50°
Elbow	10°

### Head model improvement and validation

The baseline PIPER head model was first published as a 3YO^36^. The head model has later been normalized to a baseline of a 6YO and released under an open source license together with the whole body PIPER child model. The head model in this study is further improved compared with the release version: The tentorium geometry now is updated to be more anatomically accurate; the porous skull bone is meshed with two layer hexahedral elements instead of one layer; the dura mater and pia mater incorporate nonlinear and viscoelastic properties; nonlinear models are used for the scalp. Age-dependent skull properties are also included. Table 2 summarizes the material properties used in the head model. More detailed description of head model improvement especially age-dependent material parameters are found in Supplementary Methods.

**Table 2:** Summary of material properties used in the head model.

Tissue	Material constants	*Density (kg/m*^3^)	Poisson’s ratio
Brain	*µ*_1_ = 53.8 *Pa*, *α*_1_ = 10.1, *µ*_2_ = −120.4 *Pa*, *α*_2_ = −12.9	1040.0	~0.5
CSF	*K* = 2.1 *GPa*	1000.0	0.5
Scalp connective tissue	*µ*_1_ = 1.30 × 10^4^ *Pa*, *α*_1_ = 24.2	1133.0	~0.5
Scalp adipose tissue	*µ*_1_ = 3.99 × 10^3^ *Pa*, *α*_1_ = 8.8	1133.0	~0.5
Dura mater,falx, tentorium	Hyperviscoelastic	1133.0	0.499
Pia mater	Hyperviscoelastic	1133.0	0.499
Skull	Age-dependent linear elastic*	2000.0	0.22
Suture	Age-dependent linear elastic*	1500.0	0.22

*See Supplementary Methods for details.

The performance of global impact kinematics is assessed by comparing the model predictions of a 1.5YO and 6YO with those from cadaveric head tests of similar ages reported in Loyd^37^. NISE correlation score (CS)^38^ is used to quantify the agreement (details found in Supplementary Validation Results). In addition, brain tissue response in terms of relative skull-brain motion predicted from the 18YO PIPER head model is compared with measurements by Hardy *et al*.^39^. The performances of the predicted relative skull-brain motion for three different impacts comparing with experimental data are quantified by both NISE and CORA using the same protocol presented in a previous study^40^. Only a summary of the biofidelity rating for skull-brain motion is listed in Table 3 and further details are found in Supplementary Validation Results.

**Table 3:** Biofidelity rating derived from NISE and CORA analysis for relative skull-brain motion.

Age	Test	R_NISE	R_CORA
18YO	C288-T3	7.82	5.27
	C380-T4	8.62	6.93
	C380-T5	7.95	5.85

The performance of the PIPER scaled 18YO is comparable with the KTH isotropic model (KTH ISO reported earlier^40^ with CORA ratings of 5.01, 5.23. 6.24 for C288-T3, C380-T4 and C380-T5 respectively. However, as earlier studies indicated, child head cannot be considered as a smaller version of an adult head due to the anatomies, similarly, the scaled 18YO from 6YO directly cannot represent an adult head. Nevertheless, due to the paucity of pediatric data of brain response, the approach done in this study provides a reference of the model performance, but not an intention to achieve a high rating.

### Playground modelling

**Figure 7:**
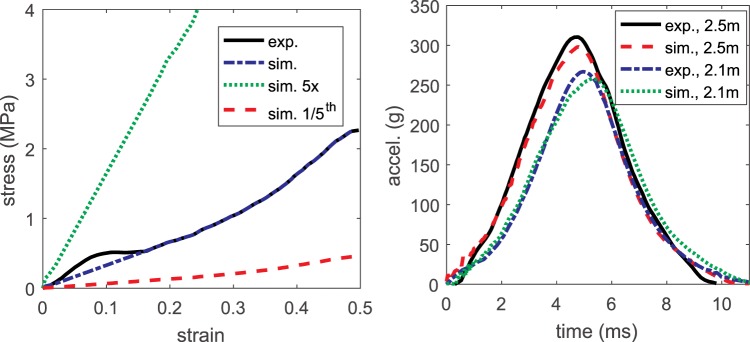
Stress–strain curve of rubber-composite specimens reported in a previous experimental study compared with the numerically implemented model of the baseline, stiffer and softer material (left). Simulated headform acceleration curves when dropped from heights of 2.1 m and 2.5 m are compared with the experimental data (right).

Tiles made of rubber composite are commonly used in playgrounds as protective surface material. In an earlier study^41^, the constitutive behaviour of a typical playground rubber-composite material was measured under an impactor of 4.86 kg with impact velocity of 6.2 m/s – similar as in the testing standard. The stress-strain curve is replotted (Fig. 7, left) and a material model *MAT_SIMPLIFIED_RUBBER in LS-Dyna is used to model the constitutive behaviour of the rubber composite defined by the stress-strain curve from the experiment. This material requires a normal stress-strain curve to define its loading behaviour. The unloading behaviours governed by hysteretic unloading and shape factor representing energy dissipation are set to 0.5 and 2.0 respectively in the model. The material performance is validated by simulating a drop test of a headform using the same setup as in the experiment from a height of 2.5 m and 2.1 m. The current implemented playground model results in accelerations curves close to the ones reported in the experimental tests^41^ (Fig. 7, right). This material model will be referred to as a baseline playground material.

To numerically evaluate the protective performance of playground with different stiffness, a stiffer and softer material is implemented by scaling the stress-strain curves to be 5 times and 1/5^th^ of the baseline respectively. Critical height is defined as the highest theoretical drop height from which a surface meets the impact attenuation performance criterion (HIC = 1000). The critical heights are determined by simulating drop of headform from different heights and paired with the obtained HIC, which are found to be 1.59 m, 0.79 m and 3.26 m for the baseline, stiffer and softer playground material respectively.

### Loading conditions

**Figure 8:**
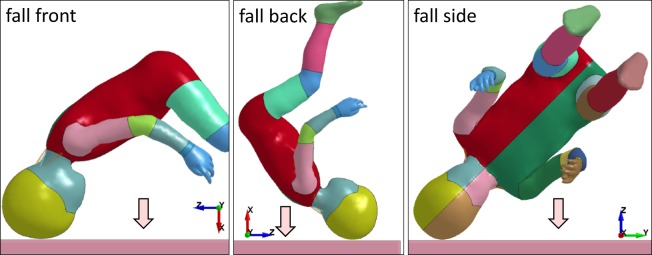
Simulation of falls from 1.59 m to a playground at front, back and side of the head, illustrated with a 3YO model. The same position applies for all ages.

Studies suggest that regardless of fall height, children tend to land on their heads after falling from a standing position and rotate during fall onto their heads while adults tend to land foot or side first^4^. Therefore, head-first impacts are simulated by rotating the positioned models of all ages to three different impact directions including front, side and back (Fig. 8). An initial velocity of 5.59 m/s is prescribed to the whole body to simulate a free fall from a critical height of 1.59 m for the baseline playground material. The same velocity is also used for the stiffer and softer playground. *CONTACT_AUTOMATIC_SURFACE_TO_SURFACE card in LS-Dyna was used to simulate the contact between the child and the playground.

### Evaluation of results

A second order (2-pole) Butterworth filter (once forwards and once backwards to avoid phase shift in the filtered data) with a cutoff frequency of 2077.5 Hz^5^ is recommend to be applied to the acceleration time-history curves prior to HIC calculation, which is implemented accordingly to a recommended algorithm by ASTM. The self-implemented filter is then verified using the ASTM data. The filtered res.lin.accel. curves are then used to calculate HIC values using the following equation:

$${\rm{HIC}}=\mathop{\max }\limits_{{{t}}_{1},{{t}}_{2}}\{({{t}}_{2}-{{t}}_{1}){[\frac{1}{({{t}}_{2}-{{t}}_{1})}{\int }_{{{t}}_{1}}^{{{t}}_{2}}a(t)dt]}^{2.5}\}$$

The HIC is the maximum value over the critical time period *t*_1_ to *t*_2_. HIC15 and HIC36 are defined by limiting *t*_2_ − *t*_1_ < 15 ms and 36 ms respectively, while HIC unlimited is defined without any limiting range. Current ASTM playground testing standard adopts HIC unlimited although both HIC15 and HIC36 have been used in automotive industry^8^. To investigate the difference, HIC15, HIC36 and HIC unlimited are all calculated, however, throughout the text HIC indicates HIC unlimited unless specified.

Linear and angular accelerations and angular velocities are extracted from the accelerometer implemented at the C.G. of the head filtered by a Butterworth filter with cut off frequency of 180 Hz (see a previous study^15^ for a more detailed description of the head accelerometer implementation). 95^th^ percentile maximum 1^st^ principal Green-Lagrangian (G-L) strain (referred to as MPS) is extracted in the brain following previous studies to avoid potential numerical issues^42,43^. Maximum von-Mises (v-M) stress in the skull bone during the entire impact is also extracted.

## Electronic supplementary material

Supplementary Material

Animation Fall Front

Animation Fall Back

Animation Fall Side

## Data Availability

The PIPER scalable child human body model (HBM), the PIPER tool are all released by the PIPER project under an open-source license (available at piper-project.org/downloads). The updated version of the PIPER HBM used in this study is available from https://gitlab.inria.fr/piper/child/.
