# Supplementary material for: Improved safety standards are needed to better protect younger children at playgrounds
Source: Sci Rep. 2018 Oct 10;8:15061. doi: 10.1038/s41598-018-33393-z (PMC6180095; doi:10.1038/s41598-018-33393-z)
Supplement: Supplementary file 1 — Supplementary Material [file 41598_2018_33393_MOESM1_ESM.pdf]

Supplementary Material

Improved safety standards are needed to better protect younger children at playgrounds

Xiaogai Li,[1] **Svein Kleiven**[1*]

[1]Division of Neuronic Engineering, Department of Biomedical Engineering and Health Systems, KTH Royal Institute of Technology, Huddinge 141 52, Sweden
*Correspondence: sveink@kth.se

Supplementary Material Summary

1. Supplementary Methods on Head Model Improvement
More detailed description of head model improvement regarding scalp, dura and pia, as well as age-dependent material properties for the skull and suture.

2. Supplementary Results
Influence of playground stiffness on time-history curves of res.lin.accel.; influence of playground stiffness on global impact kinematics.

3. Supplementary Validation Results of the Head Model
Validation results of the newly improved head model both global kinematics and relative brain-skull motion.

4. Supplementary Videos
Animations of impact kinematics during the simulated entire fall impact onto the baseline playground material illustrated with a **3YO fall front, back and side respectively.**

1. Supplementary Methods on Head Model Improvement

1.1 Scalp

A 1st order Ogden hyperelastic model is used for scalp model incorporating hyperplastic and viscoelastic behaviour. The scalp contains two layers, outer layer representing the skin - a dense connective tissue and inner layer an adipose tissue layer. Material parameters are adopted from an earlier study[1] with parameter values presented in Giordano et al. (2017)[2].

1.2 Dura and pia mater

A 1st order Ogden hyperelastic constitutive model is used for dura mater, and the parameters are fitted to include the non-linear elasticity described by van Noort et al. (1981)[3], as well as the high frequency relaxation moduli determined by Wilcox et al. (2003)[4]. The experimental data also provides a different stiffness in tension observed for dura mater[3]. To obtain the material constants, the iterative least-square algorithm of Levenberg-Marquardt is used. The resulting 1st order Ogden model shows a good correlation with the experimental data (R=0.9972) (**Suppl. Fig. 1** left).

The viscoelastic term includes a relaxation function defined as:

$$g(t) = \sum_{i=1}^{n} G_i e^{-\beta_i t}$$

Where G_i represent the shear relaxation moduli, and β_i is the decay constant. The fitted viscoelastic parameters correlate well with the experimental data of Wilcox et al. (2003) (R=0.9998) (**Suppl. Fig. 1** right). The fitted stiffness parameters G_i were then scaled according to the stiffness values of the fitted Ogden parameters, while the decay constants, β_i, were not altered. A similar procedure for pia mater is done for pia mater to obtain the Ogden parameters by fitting uniaxial stretching data of pia reported by Aimedieu and Grebe (2004)[5]. The viscoelastic constants are obtained by scaling that from the dura mater using the relationship of Ogden stiffness parameters. The final scaled parameters used in this study are listed in **Suppl. Tab. 1**.

Suppl. Tab. 1 Ogden hyperelastic and linear viscoelastic constants for dura and pia mater.

	**Dura**	**Pia**
μ_1 (Pa)	1.78e+5	1.40e+4
α_1	23.07	23.55
G_1 (Pa)	1.25e+7	1.03e+6
G_2 (Pa)	9.56e+5	7.89e+4
G_3 (Pa)	2.67e+6	2.20e+5
β_1 (1/s)	2.37	2.37
β_2 (1/s)	0.23	0.23
β_3 (1/s)	0.02	0.02

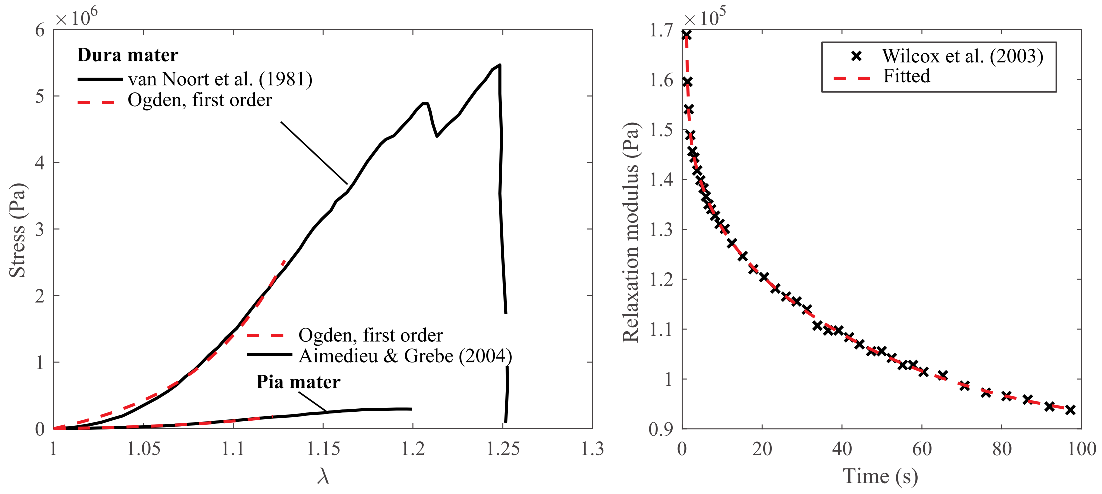

Suppl. Fig. 1 The non-linear elastic stress-strain behavior in tension for dura and pia mater (left) and high frequency shear relaxation moduli used in this study (right).

1.3 Age dependent material properties of skull and suture

As the focus of this study is on head injuries, detailed information of age dependent properties of the suture and skull are presented here. The infant skull has grain fibre patterns that are clearly visible to naked eyes at birth[6,7], causing a larger stiffness in the fibre direction confirmed by mechanical tests[8,9], being stiffer in the parallel-to-fiber direction than the perpendicular-to-fiber direction. Infant skull changes dramatically during early infancy, the grain fibre patterns becomes almost invisible in six-month-olds[10], the anisotropy ratio further decreases to 1.25 in six-year-olds[8] and becomes isotropic in adults. Meanwhile, the skull bone gets stiffer with age, ranging from a few hundred MPa in infants[6] up to several GPa in 6-year-olds[11]. Therefore, material modelling for infant skull at different ages should reflect the two biological growth factors, decrease with anisotropy and an increase in stiffness. In an earlier study, anisotropic ratio for infants was fitted based on experimental data 554 specimens from 16 foetal calvaria (20-42-week gestation)[8].

Experimental data reported in the literature for skull bone is collected and fitted to get age-dependent stiffness values for 1.5 YO to 18 YO, including infant skull data (0-18 months) from Coats and Margulies (2006)[6] and McPherson and Kriewall (1980)[9], Thibault (1999)[12] and Wang et al. (2014)[13]; 6 YO from Davis et al. (2012)[11], 7 YO from Thibault (1999)[12], as well as adult value aging from 25-95 YO from Wood et al. (1970)[14]. For infants, average stiffness of parallel-to-fiber E_1 and perpendicular-to-fiber directions (E_2) is used in the data fitting. For studies only measured E_2, the anisotropic ratio curve with age presented earlier is used to calculate stiffness along the parallel-to-fiber direction (E_1)[1], and averaged value of used in the data fitting with a one term logarithm function assuming isotropic from 1.5 YO needed in this study (R = 0.85) (**Suppl. Fig. 2**, left).

Age-dependent suture properties is obtained by fitting the data of newborn to 12 month-old from Coats and Margulies (2006)[6], a 18MO from Wang et al. (2014)[13], and a 6YO from Davis et al. (2012)[11] using a linear function (R=0.97) (**Suppl. Fig. 2,** right). Sutures for only 1.5YO and 3YO are modelled as in older ages sutures grow to bone.

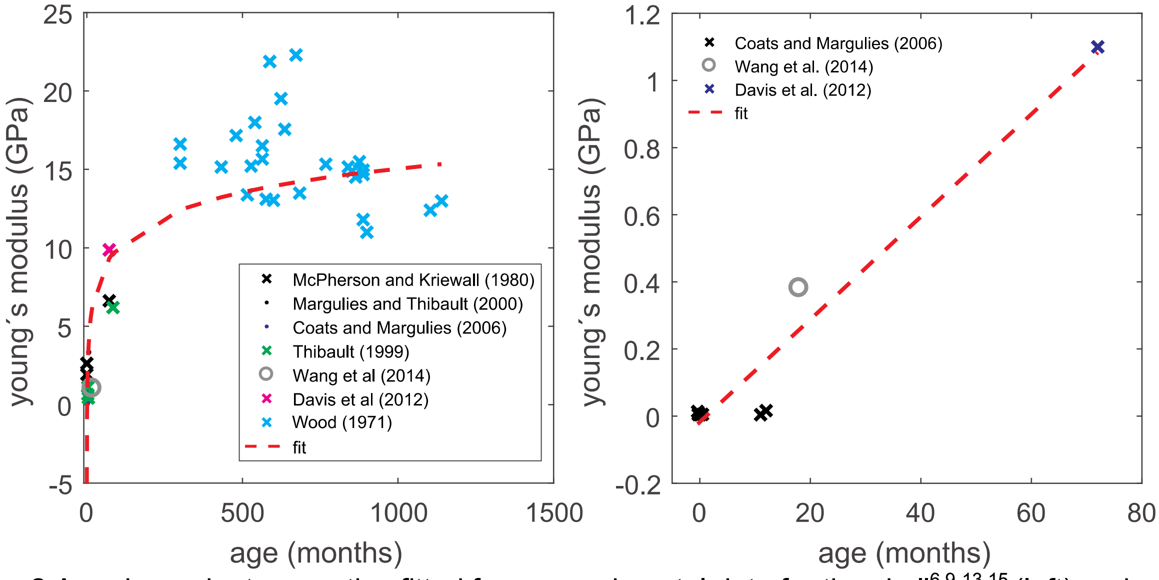

Suppl. Fig. 2 Age dependent properties fitted from experimental data for the skull[6,9-13,15] (left) and sutures[6,11,13] (right).

2. Supplementary Results

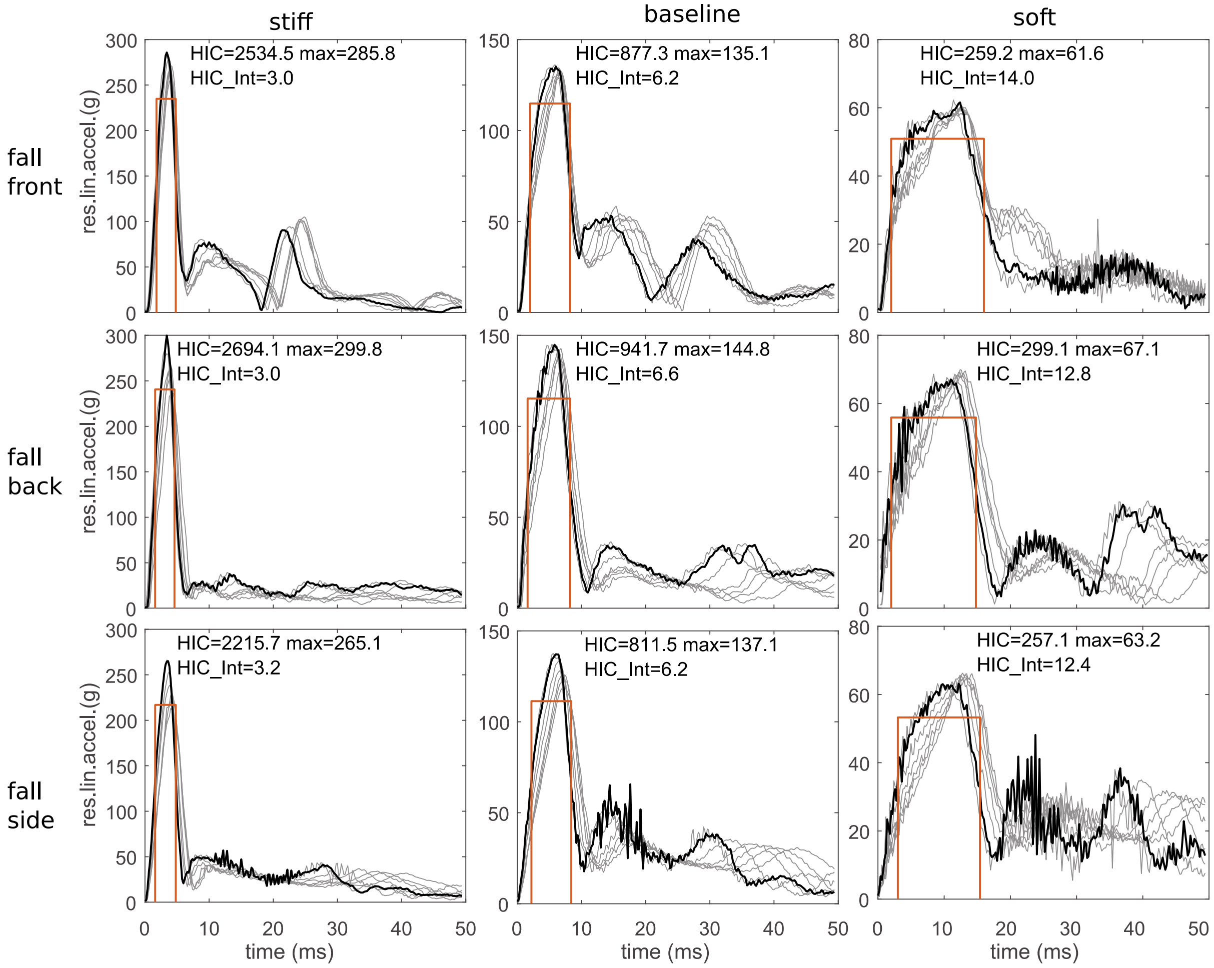

Suppl. Fig. 3 Influence of playground stiffness on time-history curves of res.lin.accel. All ages are plotted but only the 3YO is highlighted with black solid line and HIC calculated for fall front (row 1), fall back (row 2) and fall side (row 3) at a stiff, baseline and softer material playground material.

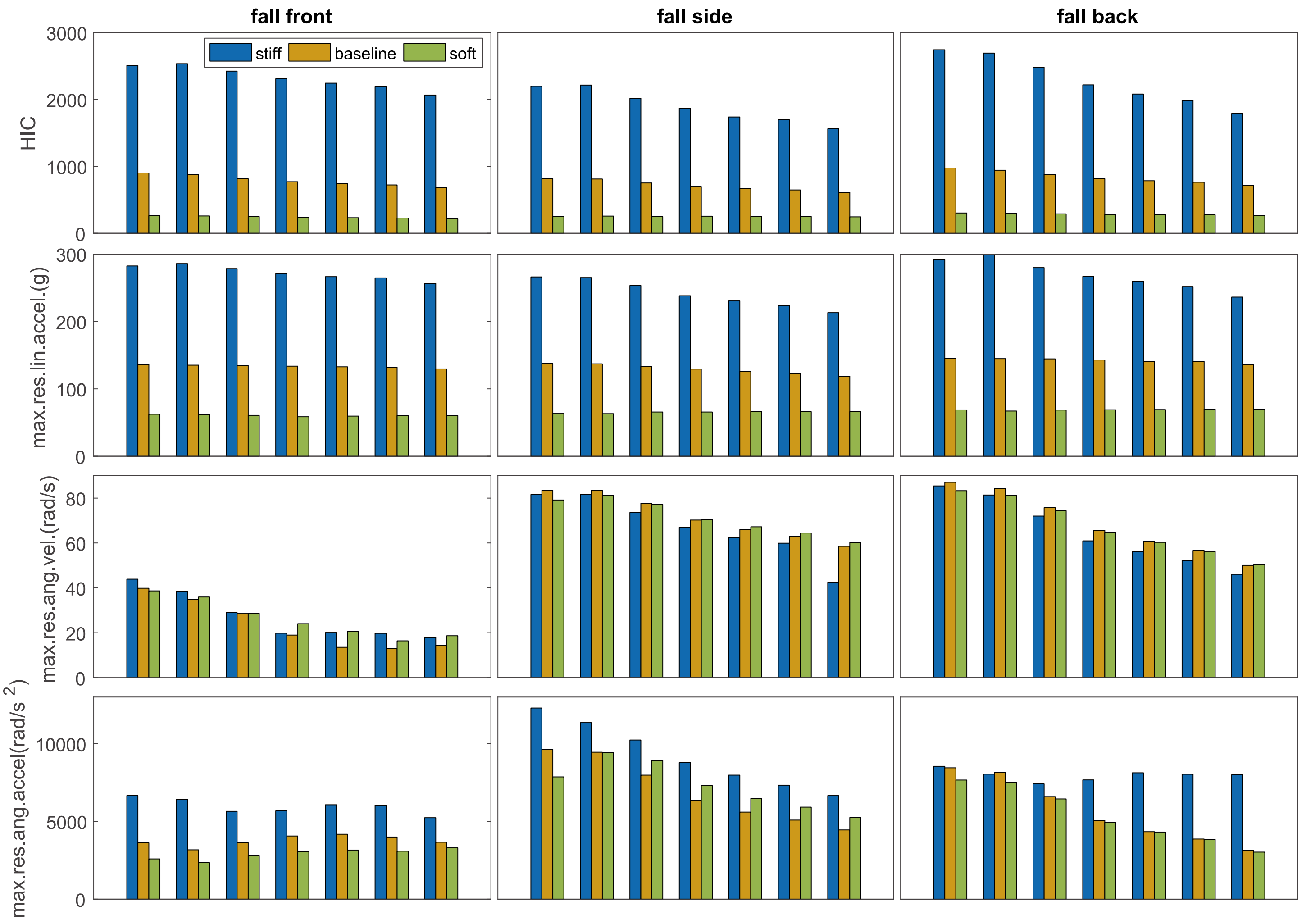

Suppl. Fig. 4 Bar plot shows the influence of impact position and playground stiffness on HIC (row 1), maximum resultant linear acceleration (row 2), maximum resultant angular velocity (row 3) and maximum resultant angular acceleration (row 4).

3. Supplementary Validation Results of the Head Model

Loyd (2011)[16] performed drop tests of PMHS heads of different ages, including a 22 months old (MO) (specimen P17F) and a 9YO (specimen P18M) at different locations. The entire time-history curves of resultant linear accelerations are available for the 9YO at five impact locations dropped from 15 cm and 30 cm, while for the 22MO, only peak values and duration is provided at three impact locations. The performances of the 1.5YO and 6YO PIPER head models are compared to above experimental data at similar ages. To account for potential head size difference due to slight age difference, the 1.5YO head model is scaled according to the reported head length and width of the 22MO, similarly the 6YO PIPER head model is scaled accordingly to the 9YO. The models are positioned to the same impact angles as in the experiment prior to impact.

Further, to evaluate the model performance of tissue response, the baseline head model is scaled to an adult (18YO) using the PIPER tool, and then further scaled according to the head length and width reported in Hardy et al. (2007)[17]. In all cases, the translational accelerations and rotational velocity are applied to the center of gravity of the head. Model predictions of skull-brain relative motion are compared to three representative cases (sagittal impact C288-T3, coronal impact C380T-4 and lateral impact C380-T5). To identify the target positions and extract characteristic curves, the PIPER 18YO is scaled to match the experimental post-mortem head and the coordinates were expressed in relations to the head center. CORA and NISE scores are calculated to

quantify the model performance on brain motion following the same procedure published earlier[18], and the results are presented in Suppl. Tab. 2 - Tab. 7.

3.1 Validation of drop tests

NISE correlation scores (CS) are calculated to quantify the agreement between model predictions and experiments. CS values range from 0 to 100, with values between 86 ≤ CS < 100 classified as *excellent* and 65 ≤ CS < 86 as *good* according to a biofidelity rating (see details in an earlier study[1]. The acceleration–time impact curves for the 6YO generally agree with the measurements, achieving a *good* to *excellent* biofidelity rating.

For the 1.5YO, notable differences are observed in peak head acceleration between model prediction and the experiment, especially the occiput and vertex impacts. It should be noted that the 22MO fractured on the 15 cm vertex impact in the experiment, may cause lower peak accelerations at subsequent impacts with a fractured skull, while no failure in the skull is included in the current models, as fractures are unlikely to occur at a drop height of 30 cm[16].

3.1.1 Validation of the 6YO

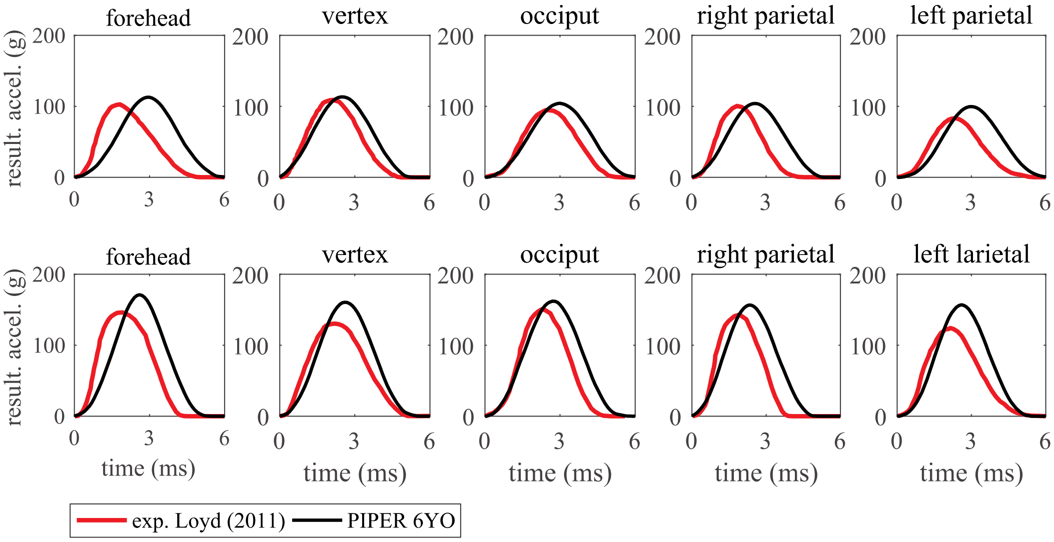

Suppl. Fig. 5 Simulated and experimental head acceleration–time curves for the impacts at five different locations for the 6YO PIPER head model with a drop height of 15 cm (upper row) and 30 cm (lower row).

Suppl. Tab. 2 NISE analysis for drop tests of the PIPER 6YO head.

Drop	$CS_{N\text{-}phase}$	$CS_{N\text{-}amp}$	$CS_{N\text{-}shape}$	Average
**15 cm drop**				
Forehead15	60.63	98.66	79.29	79.53
Vert15	87.73	99.55	91.29	92.86
Occiput15	85.97	98.82	89.93	91.57
RParietal15	75.29	98.72	87.89	87.30
LParietal15	75.94	96.63	85.07	85.88
**30 cm drop**				
ForeHead30	76.43	99.31	86.57	87.44
Vert30	88.03	98.49	90.86	92.45
Occiput30	87.47	98.91	91.75	92.71
RParietal30	83.29	98.78	91.08	91.05
LParietal30	85.96	97.01	90.12	91.03

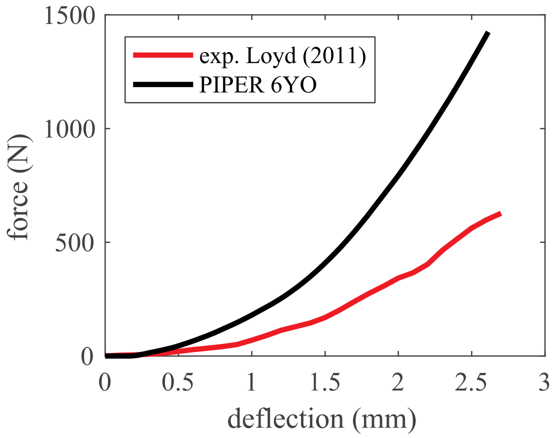

Suppl. Fig. 6 Comparison of experimental (data referring to P17F) and simulated force deflection curves for the compression test (0.3/s).

3.1.1 Validation of the 1.5YO

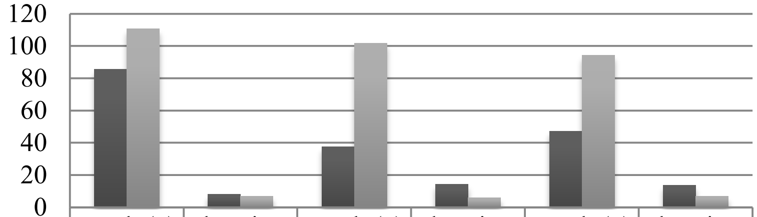

	forehead		occiput		vertex	
	accel. (g)	duration	accel. (g)	duration	accel. (g)	duration
exp. Loyd (2011)	85.6	8.2	37.6	14.2	47.2	13.6
PIPER 18M	110.6	6.8	101.9	6	94.4	6.9

Suppl. Fig. 7 Simulated and experimental peak head acceleration and impact duration at different impact locations for the 1.5YO head model with a drop height of 15 cm.

3.2 Validation of brain motion

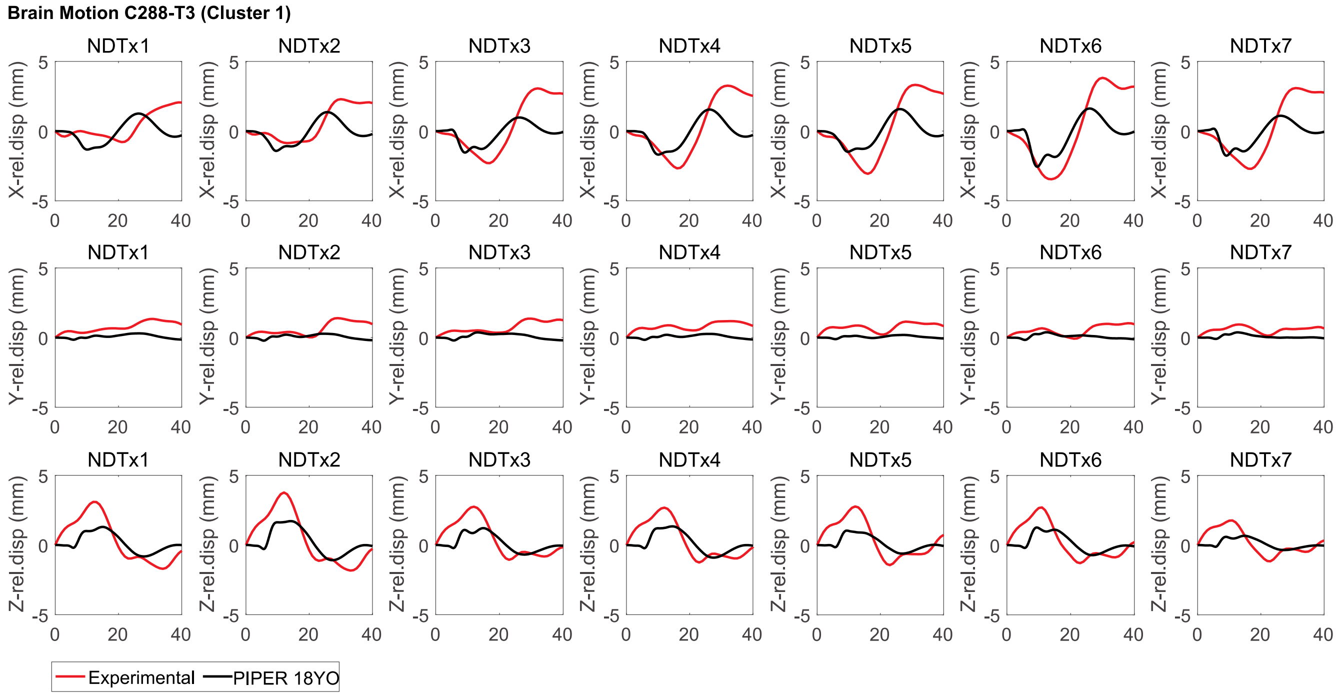

Suppl. Fig. 8 Comparison between experimental and simulated brain motion for the experiment C288-T3 at cluster 1 (NDT1-NDT7).

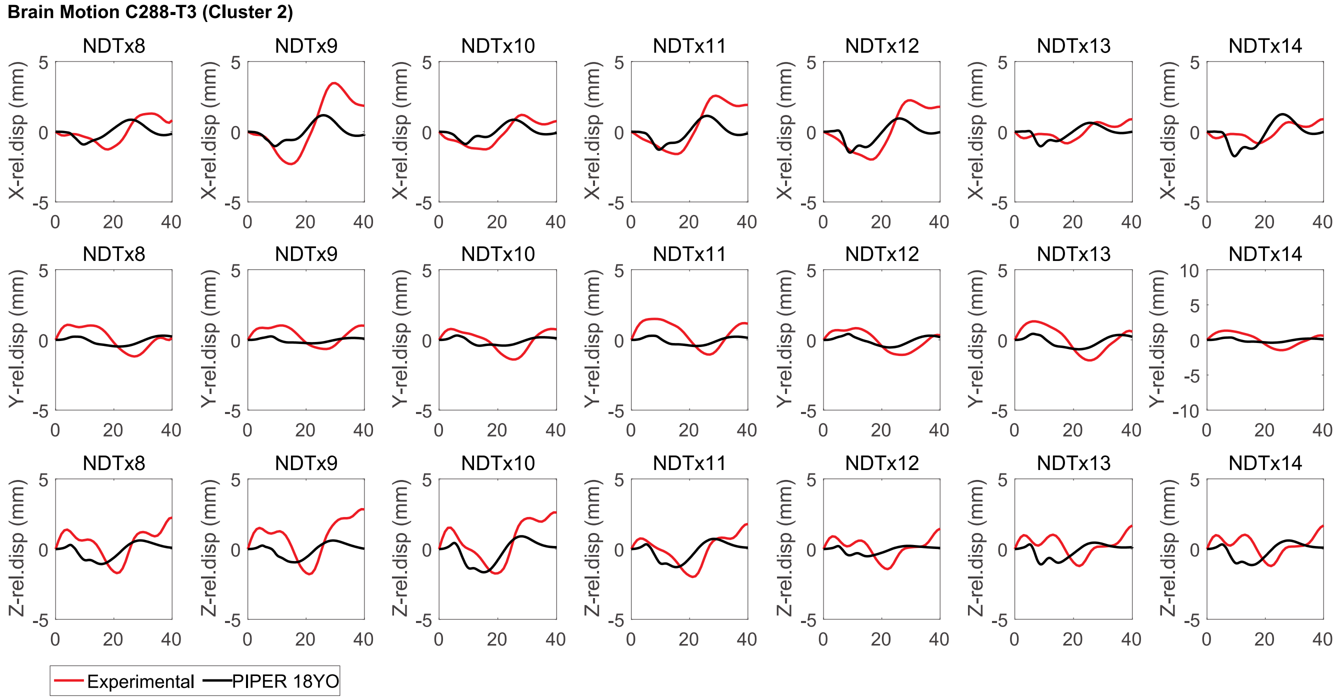

Suppl. Fig. 9 Comparison between experimental and simulated brain motion for the experiment C288-T3 at cluster 2 (NDT8-NDT14).

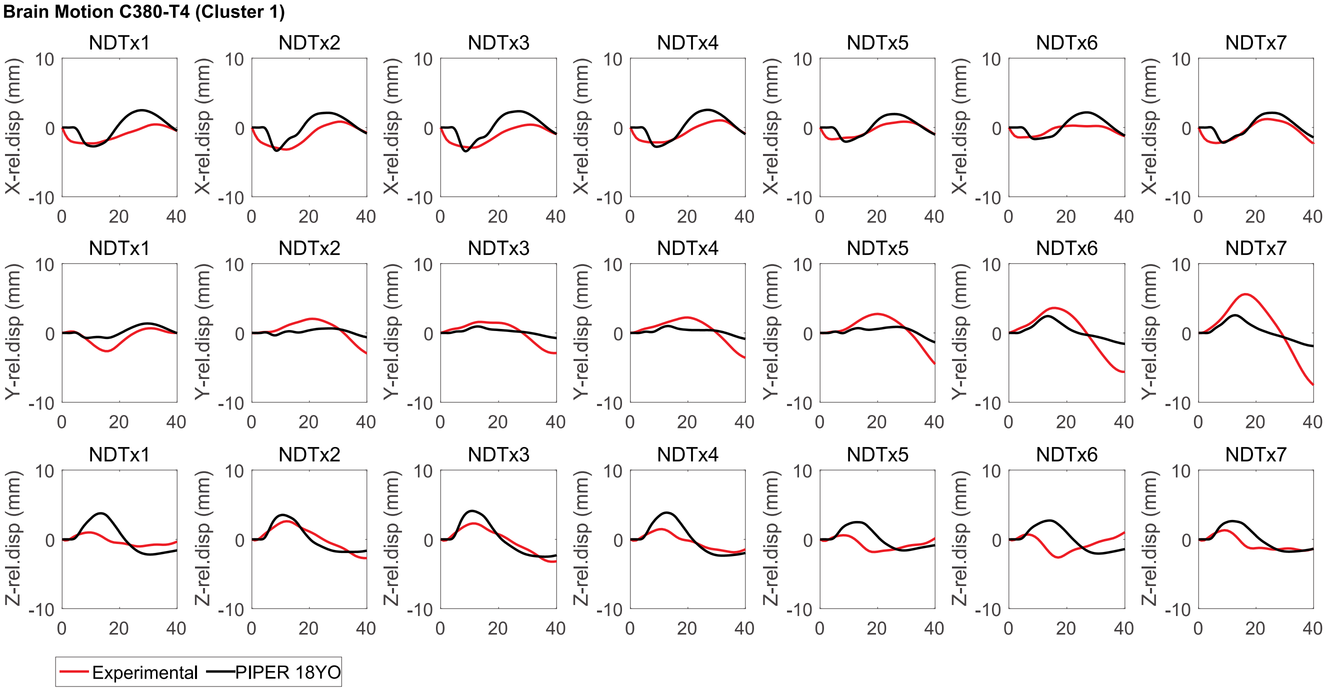

Suppl. Fig. 10 Comparison between experimental and simulated brain motion for the experiment C380-T4 at cluster 1 (NDT1-NDT7).

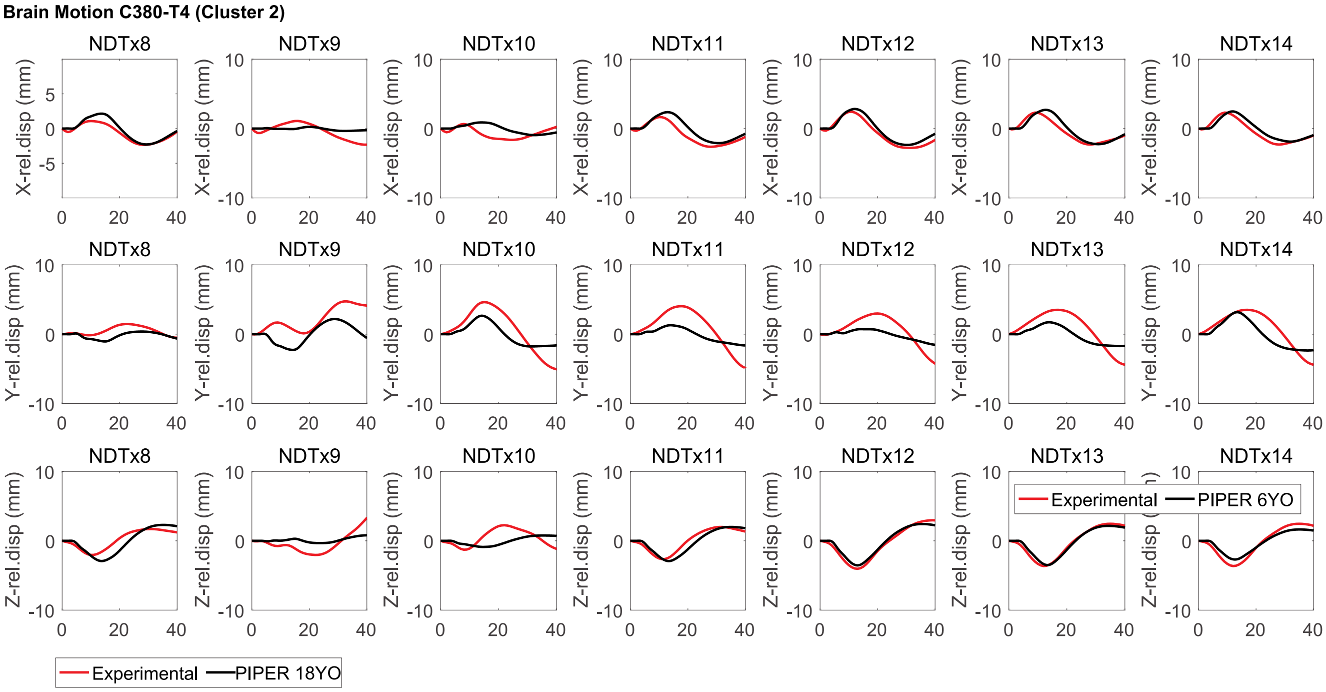

Suppl. Fig. 11 Comparison between experimental and simulated brain motion for the experiment C380-T4 at cluster 2 (NDT8-NDT14).

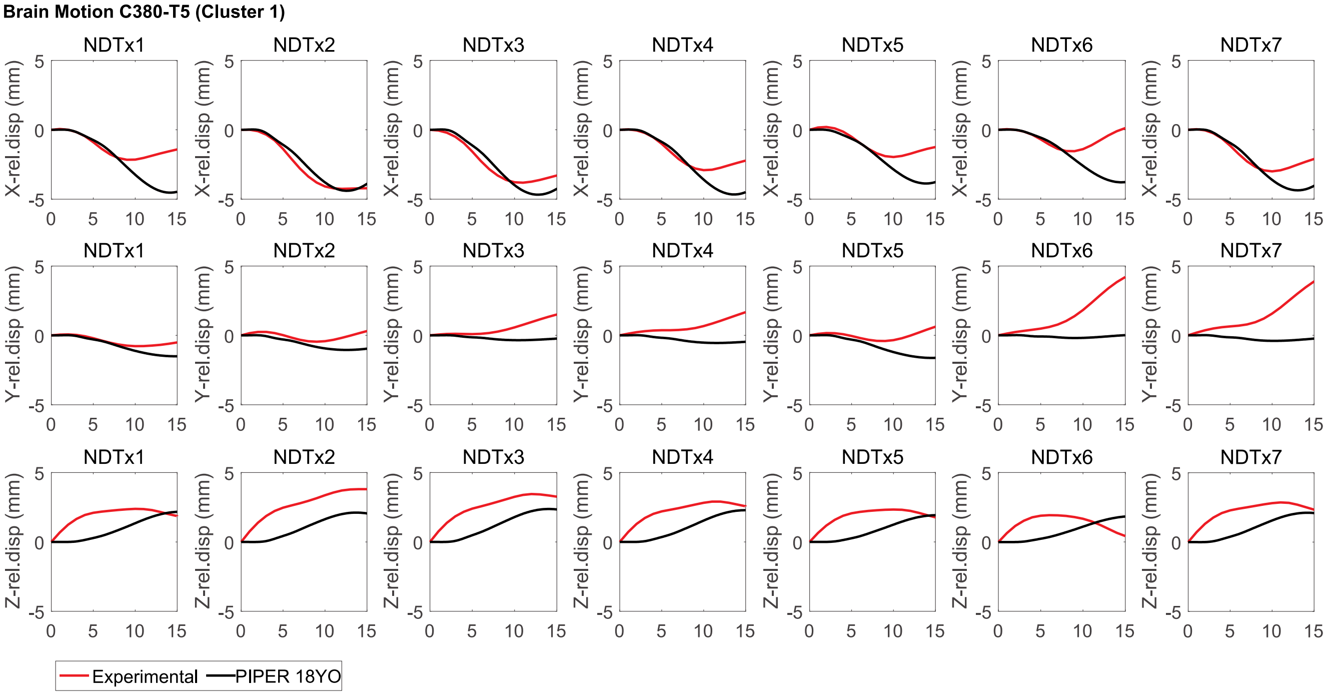

Suppl. Fig. 12 Comparison between experimental and simulated brain motion for the experiment C380-T5 at cluster 1 (NDT1-NDT7).

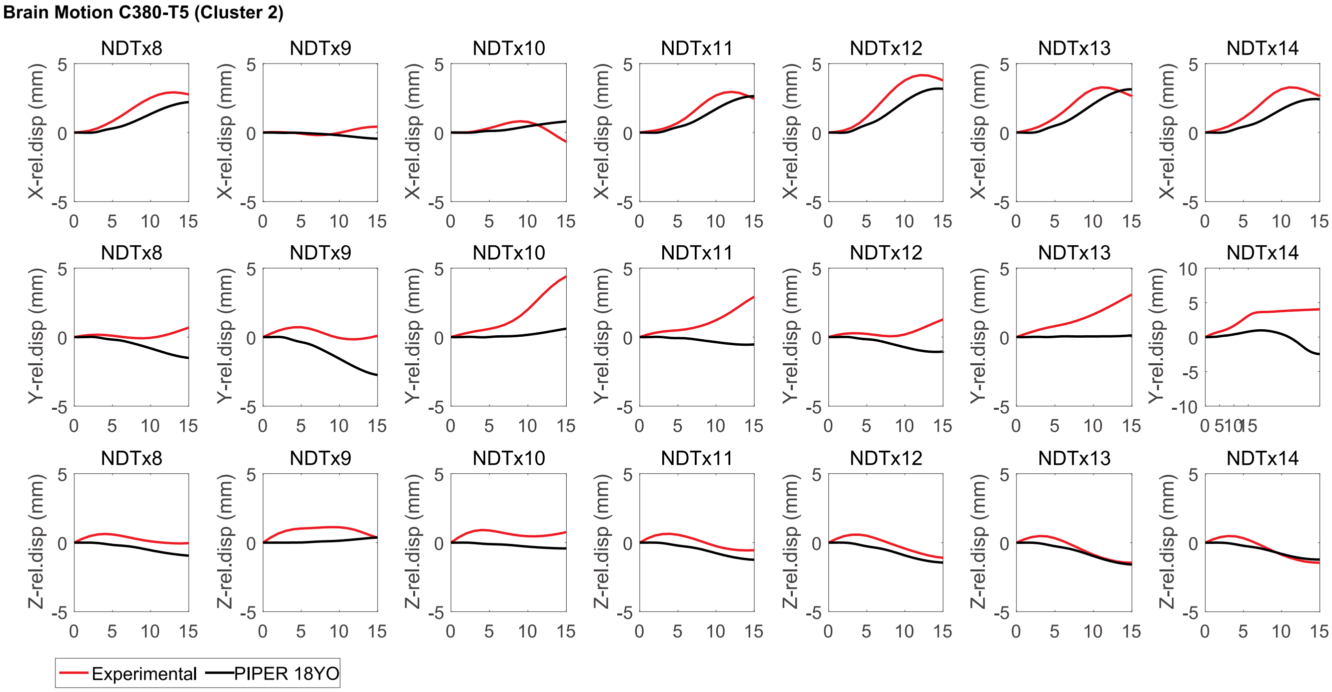

Suppl. Fig. 13 Comparison between experimental and simulated brain motion for the experiment C380-T5 at cluster 2 (NDT8-NDT14).

Suppl. Tab. 3 NISE analysis for relative skull-brain motion for case C288-T3.

NDT	AXIS	NISE Phase	NISE amplitude	NISE Shape	R
01	X	0.0202	0.0045	0.8805	6.98
01	Y	-0.0127	0.3163	0.5125	7.28
01	Z	0.0877	0.2743	0.0532	8.62
02	X	-0.0788	0.0365	0.6800	7.87
02	Y	0.0227	0.1821	0.7082	6.96
02	Z	0.0865	0.1924	0.0496	8.91
03	X	-0.0568	0.1511	0.5386	7.89
03	Y	-0.0025	0.1121	0.7963	6.98
03	Z	0.1646	0.2445	-0.0192	8.70
04	X	-0.0741	0.1082	0.5447	8.07
04	Y	0.0266	0.3835	0.4382	7.17
04	Z	0.1887	0.1690	-0.0099	8.84
05	X	-0.0656	0.1539	0.4873	8.08
05	Y	0.0460	0.5918	0.2336	7.10
05	Z	0.1134	0.3328	0.0075	8.49
06	X	-0.0484	0.1589	0.3608	8.43
06	Y	0.0161	0.1351	0.7521	6.99
06	Z	0.2617	0.2230	-0.0503	8.55
07	X	-0.0555	0.1668	0.4600	8.10
07	Y	0.0139	0.3673	0.3498	7.56
07	Z	0.3602	0.3993	-0.1221	7.88
08	X	-0.1259	0.0179	0.8532	7.52
08	Y	0.4360	0.3723	-0.0205	7.37
08	Z	0.1332	0.1178	0.4580	7.64
09	X	-0.0563	0.2316	0.4612	7.88
09	Y	0.1515	0.4380	0.2355	7.25
09	Z	0.0779	0.2358	0.4682	7.39
10	X	-0.0715	0.0995	0.4035	8.56
10	Y	-0.0547	0.2122	0.4387	8.01
10	Z	-0.0736	0.0743	0.5233	8.25
11	X	-0.0499	0.1618	0.4147	8.24
11	Y	0.2404	0.4479	0.1291	7.28
11	Z	-0.1138	0.0382	0.5869	8.30
12	X	-0.0547	0.1524	0.3877	8.38
12	Y	0.0310	0.1855	0.2907	8.31
12	Z	0.1830	0.1611	0.5615	6.98
13	X	-0.0879	0.0024	0.6625	8.08
13	Y	0.1998	0.2353	0.1629	8.01
13	Z	0.5688	0.0352	0.5703	6.09
14	X	-0.0792	0.0480	0.5831	8.16
14	Y	0.0580	0.3837	0.2675	7.64
14	Z	0.6180	0.0164	0.5109	6.18

Suppl. Tab. 4 NISE analysis for relative skull-brain motion for case C380-T4.

NDT	AXIS	NISE Phase	NISE amplitude	NISE Shape	R
01	X	-0.0095	0.0090	0.4829	8.39
01	Y	-0.0477	0.0581	0.4968	8.31
01	Z	0.1815	0.3975	-0.0002	8.07
02	X	-0.0322	0.0050	0.3932	8.78
02	Y	0.0395	0.4293	0.1474	7.95
02	Z	-0.0385	0.0075	0.1294	9.67
03	X	-0.0155	0.0001	0.4319	8.61
03	Y	-0.0025	0.3876	0.1155	8.33
03	Z	-0.0216	0.0316	0.0953	9.65
04	X	-0.0112	0.0204	0.2837	9.02
04	Y	-0.0064	0.4016	0.1361	8.23
04	Z	0.0403	0.1636	0.0740	9.07
05	X	-0.0006	0.0197	0.2450	9.12
05	Y	-0.0090	0.3888	0.0967	8.41
05	Z	0.5954	0.0425	0.1856	7.26
06	X	0.2698	0.0910	0.1624	8.26
06	Y	-0.0263	0.2665	0.1513	8.70
06	Z	1.2626	0.0481	0.0385	5.50
07	X	0.0349	0.0003	0.1748	9.30
07	Y	-0.0410	0.3260	0.1734	8.47
07	Z	0.2299	0.0348	0.1427	8.64
08	X	0.0485	0.0051	0.0379	9.69
08	Y	0.1731	0.0279	0.7079	6.97
08	Z	0.1763	0.0466	-0.0439	9.40
09	X	-0.0056	0.5475	0.1970	7.54
09	Y	-0.0405	0.0652	0.6355	7.80
09	Z	-0.0312	0.3783	0.3205	7.77
10	X	0.7140	0.0884	0.0335	7.21
10	Y	-0.0530	0.1642	0.2215	8.89
10	Z	0.8604	0.2412	-0.0438	6.47
11	X	0.1154	0.0046	0.0343	9.49
11	Y	-0.0530	0.2760	0.2920	8.28
11	Z	0.1099	0.0015	-0.0368	9.75
12	X	0.0250	0.0037	0.0222	9.83
12	Y	-0.0589	0.2970	0.2772	8.28
12	Z	-0.0052	0.0105	0.0146	9.93
13	X	0.1165	0.0018	-0.0374	9.73
13	Y	-0.0611	0.1645	0.2951	8.67
13	Z	0.0183	0.0040	-0.0088	9.95
14	X	0.1147	0.0040	-0.0366	9.73
14	Y	-0.0717	0.0342	0.2806	9.19
14	Z	0.0145	0.0614	-0.0093	9.78

Suppl. Tab. 5 NISE analysis for relative skull-brain motion for case C380-T5.

NDT	AXIS	NISE Phase	NISE amplitude	NISE Shape	R
01	X	0.1346	0.1759	-0.0784	9.23
01	Y	0.0697	0.1495	-0.0367	9.39
01	Z	0.3077	0.1274	-0.1726	9.13
02	X	0.0032	0.0019	0.0014	9.98
02	Y	0.2426	0.2775	0.1791	7.67
02	Z	0.0435	0.2391	0.0023	9.05
03	X	0.0418	0.0053	-0.0252	9.93
03	Y	0.5091	0.0000	1.0000	4.97
03	Z	0.0876	0.1647	-0.0316	9.26
04	X	0.0711	0.0667	-0.0385	9.67
04	Y	0.7077	0.0000	1.0000	4.31
04	Z	0.1569	0.1473	-0.0704	9.22
05	X	0.0939	0.1712	-0.0503	9.28
05	Y	0.4345	0.3595	0.2327	6.58
05	Z	0.2957	0.1657	-0.1758	9.05
06	X	0.4293	0.3996	-0.2682	8.13
06	Y	0.0616	0.0029	0.9968	6.46
06	Z	0.7539	0.0876	-0.4332	8.64
07	X	0.0865	0.0484	-0.0546	9.73
07	Y	0.2385	0.0000	1.0000	5.87
07	Z	0.1876	0.1626	-0.1059	9.19
08	X	0.0095	0.0977	0.0097	9.61
08	Y	0.4189	0.0000	1.0000	5.27

08	Z	0.1632	0.0000	1.0000	6.12
09	X	1.3248	0.0018	0.4214	4.17
09	Y	0.0349	0.0000	1.0000	6.55
09	Z	0.2701	0.8258	-0.3233	7.42
10	X	0.9252	0.0108	-0.1903	7.51
10	Y	-0.0161	0.6989	0.0916	7.42
10	Z	0.4996	0.0000	1.0000	5.00
11	X	0.0290	0.0309	-0.0054	9.82
11	Y	0.4268	0.0000	1.0000	5.24
11	Z	-0.1124	0.0363	0.5959	8.27
12	X	0.0141	0.0697	-0.0014	9.73
12	Y	0.8606	0.0000	1.0000	3.80
12	Z	-0.1387	0.0306	0.3707	9.12
13	X	0.0588	0.0204	-0.0229	9.81
13	Y	-0.0040	0.8520	0.0871	6.88
13	Z	-0.0836	0.0017	0.1547	9.76
14	X	0.0517	0.0884	-0.0238	9.61
14	Y	-0.0272	0.5053	0.0683	8.18
14	Z	-0.0870	0.0094	0.1629	9.72

Suppl. Tab. 6 Results of the CORA analysis for relative skull-brain motion for case C288-T3.

NDT	AXIS	V	G	P	C2
01	X	0.532	0.453	0.565	5.17
01	Y	0.683	0.031	0.565	4.27
01	Z	0.842	0.179	0.828	6.16
02	X	0.545	0.321	0.697	5.21
02	Y	0.494	0.035	0.565	3.65
02	Z	0.845	0.249	0.894	6.63
03	X	0.635	0.134	0.763	5.11
03	Y	0.404	0.048	0.565	3.39
03	Z	0.897	0.214	0.828	6.46
04	X	0.698	0.196	0.763	5.52
04	Y	0.536	0.026	0.565	3.76
04	Z	0.885	0.291	0.828	6.68
05	X	0.729	0.154	0.763	5.49
05	Y	0.578	0.013	0.828	4.73
05	Z	0.882	0.147	0.828	6.19
06	X	0.709	0.198	0.894	6.00
06	Y	0.274	0.106	0.565	3.15
06	Z	0.908	0.240	0.763	6.37
07	X	0.683	0.150	0.828	5.54
07	Y	0.575	0.058	0.894	5.09
07	Z	0.966	0.135	0.697	5.99
08	X	0.515	0.286	0.828	5.43
08	Y	0.664	0.068	0.631	4.55
08	Z	0.611	0.217	0.631	4.86
09	X	0.772	0.093	0.763	5.42
09	Y	0.616	0.045	0.697	4.53
09	Z	0.609	0.079	0.697	4.62
10	X	0.749	0.280	0.828	6.19
10	Y	0.771	0.118	0.763	5.50
10	Z	0.600	0.274	0.828	5.67
11	X	0.674	0.171	0.828	5.58
11	Y	0.537	0.065	0.697	4.33
11	Z	0.779	0.345	0.697	6.07
12	X	0.719	0.193	0.828	5.80
12	Y	0.882	0.198	0.763	6.14
12	Z	0.618	0.089	0.565	4.24
13	X	0.632	0.774	0.631	6.79
13	Y	0.758	0.196	0.697	5.50
13	Z	0.379	0.283	0.565	4.09
14	X	0.636	0.376	0.697	5.69
14	Y	0.771	0.070	0.697	5.13
14	Z	0.462	0.387	0.565	4.72

Suppl. Tab. 7 Results of the CORA analysis for relative skull-brain motion for case C380-T4.

NDT	AXIS	V	G	P	C2
01	X	0.456	0.684	0.894	6.78
01	Y	0.532	0.364	0.894	5.97
01	Z	0.890	0.103	0.763	5.85
02	X	0.595	0.781	0.828	7.35
02	Y	0.893	0.103	0.763	5.86
02	Z	0.895	0.784	0.960	8.80
03	X	0.504	0.979	0.960	8.14
03	Y	0.865	0.123	0.894	6.27
03	Z	0.901	0.552	1.000	8.18
04	X	0.648	0.622	0.960	7.43
04	Y	0.841	0.110	0.894	6.15
04	Z	0.892	0.240	0.894	6.75
05	X	0.671	0.625	1.000	7.65
05	Y	0.920	0.126	0.894	6.47
05	Z	0.585	0.488	0.565	5.46
06	X	0.594	0.315	0.763	5.57
06	Y	0.845	0.150	0.960	6.52
06	Z	0.426	0.514	0.565	5.02
07	X	0.771	0.907	0.894	8.57
07	Y	0.921	0.089	0.828	6.13
07	Z	0.783	0.427	0.697	6.36
08	X	0.897	0.807	0.960	8.88
08	Y	0.255	0.399	0.763	4.72
08	Z	0.983	0.491	0.763	7.46
09	X	0.755	0.032	0.960	5.82
09	Y	0.457	0.234	0.763	4.85
09	Z	0.651	0.040	0.894	5.28
10	X	0.664	0.418	0.565	5.49
10	Y	0.895	0.204	0.828	6.42
10	Z	0.678	0.226	0.565	4.90
11	X	0.888	0.890	0.828	8.69
11	Y	0.932	0.081	0.697	5.70
11	Z	0.985	0.867	0.894	9.15
12	X	0.949	0.873	0.960	9.27
12	Y	0.962	0.063	0.697	5.74
12	Z	0.986	0.753	1.000	9.13
13	X	0.979	0.857	0.894	9.10
13	Y	0.917	0.162	0.697	5.92
13	Z	0.995	0.863	0.960	9.39
14	X	0.977	0.864	0.894	9.12
14	Y	0.908	0.401	0.697	6.69
14	Z	0.996	0.504	0.960	8.20

Suppl. Tab. 8 Results of the CORA analysis for relative skull-brain motion for case C380-T5.

NDT	AXIS	V	G	P	C2
01	X	0.979	0.273	0.659	6.37
01	Y	0.977	0.325	0.842	7.15
01	Z	0.881	0.377	0.659	6.39
02	X	0.998	0.960	0.842	9.33
02	Y	0.674	0.146	0.659	4.93
02	Z	0.961	0.275	0.659	6.32
03	X	0.996	0.746	0.842	8.61
03	Y	0.001	0.421	0.659	3.60
03	Z	0.957	0.359	0.659	6.58
04	X	0.991	0.404	0.659	6.85
04	Y	3.540	0.282	1.000	4.27
04	Z	0.926	0.364	0.659	6.50
05	X	0.970	0.266	0.659	6.32
05	Y	0.712	0.054	0.659	4.75
05	Z	0.889	0.324	0.659	6.24
06	X	0.920	0.189	0.659	5.90
06	Y	0.006	0.009	0.659	2.25
06	Z	0.789	0.404	0.659	6.18
07	X	0.990	0.462	0.659	7.04
07	Y	0.000	0.041	0.842	2.95
07	Z	0.932	0.344	0.659	6.45
08	X	0.999	0.550	0.659	7.36
08	Y	0.083	0.017	0.659	2.53
08	Z	0.104	0.581	0.659	4.48
09	X	0.134	0.290	0.659	3.61
09	Y	0.115	0.093	0.842	3.50
09	Z	0.759	0.029	0.659	4.82
10	X	0.901	0.691	0.659	7.50
10	Y	0.984	0.027	0.659	5.57
10	Z	0.003	0.120	0.842	3.22
11	X	0.995	0.634	0.842	8.24
11	Y	9.260	0.090	0.842	3.11
11	Z	0.573	0.558	0.659	5.97
12	X	0.998	0.511	0.842	7.84
12	Y	0.001	0.146	0.659	2.69
12	Z	0.835	0.821	0.659	7.72
13	X	0.999	0.817	0.659	8.25
13	Y	0.957	0.001	1.000	6.53
13	Z	0.938	0.739	0.659	7.79
14	X	0.999	0.523	0.659	7.27
14	Y	0.995	0.105	0.659	5.86
14	Z	0.942	0.493	0.659	6.98

4. Reference

1. Li X, Sandler H, Kleiven S. The importance of nonlinear tissue modelling in finite element simulations of infant head impacts. *Biomech Model Mechanobiol*. 2017; **16**:823–840.
2. Giordano C, Li X, Kleiven S. Performances of the PIPER scalable child human body model in accident reconstruction. *PloS one*. 2017; **12**.
3. van Noort R, Black M, Martin T, Meanley S. A study of the uniaxial mechanical properties of human dura mater preserved in glycerol. *Biomaterials*. 1981; **2**:41–45.
4. Wilcox RK, Bilston LE, Barton DC, Hall RM. Mathematical model for the viscoelastic properties of dura mater. *J Orthop Sci*. 2003; **8**:432–434.
5. Aimedieu P, Grebe R. Tensile strength of cranial pia mater: preliminary results. *J Neurosurg*. 2004; **100**:111–114.
6. Coats B, Margulies SS. Material properties of human infant skull and suture at high rates. *J Neurotrauma*. 2006; **23**:1222–1232.
7. Holck P. What can a baby's skull withstand? Testing the skull's resistance on an anatomical preparation. *Forensic Sci Int*. 2005; **151**:187–191.
8. Kriewall TJ. Structural, mechanical, and material properties of fetal cranial bone. *Am J Obstet Gynecol*. 1982; **143**:707–714.
9. McPherson GK, Kriewall TJ. The elastic modulus of fetal cranial bone: a first step towards an understanding of the biomechanics of fetal head molding. *J Biomech*. 1980; **13**:9–16.
10. Margulies SS, Thibault KL. Infant skull and suture properties: measurements and implications for mechanisms of pediatric brain injury. *J Biomech Eng*. 2000; **122**:364–371.
11. Davis MT, Loyd AM, Shen HyH, Mulroy MH, Nightingale RW, et al. The mechanical and morphological properties of 6 year-old cranial bone. *J Biomech*. 2012; **45**:2493–2498.
12. Thibault KL, Kurtz SM, Runge CF, Giddings VL, Thibault LE. Material properties of the infant skull and application to numerical analysis of pediatric head injury. In: IRCOBI; 1999. p. 73–82.
13. Wang J, Zou D, Li Z, Huang P, Li D, et al. Mechanical properties of cranial bones and sutures in 1–2-year-old infants. *Medical science monitor: international medical journal of experimental and clinical research*. 2014; **20**:1808.
14. McElhaney JH, Fogle JL, Melvin JW, Haynes RR, Roberts VL, et al. Mechanical properties of cranial bone. *J Biomech*. 1970; **3**:495IN5497–496511.
15. Wood JL. Dynamic response of human cranial bone. *J Biomech*. 1971; **4**:IN1–IN3.
16. Loyd AM. Studies of the human head from neonate to adult: an inertial, geometrical and structural analysis with comparisons to the ATD head. Duke University; 2011.
17. Hardy WN, Mason MJ, Foster CD, Shah CS, Kopacz JM, et al. A study of the response of the human cadaver head to impact. *Stapp Car Crash J*. 2007; **51**:17–80.
18. Giordano C, Kleiven S. Development of an unbiased validation protocol to assess the biofidelity of finite element head models used in prediction of traumatic brain injury. *Stapp Car Crash J*. 2016; **60**:363–471.
